# 

**Published:** 1883-02

**Authors:** 


### Pamphlets.

Bromide of Ethyl, the most perfect anæsthetic for short, painful surgical operations. By Julian J. Chisolm, M.D., Baltimore. Reprint from the *Maryland Medical Journal.*

The Fourth Annual Report of the Board of Health of the City of Atlanta. For the year 1882. W. S. Armstrong, M.D., President; James B. Baird, M.D., Secretary.

Transactions of the Medical Society of the State of Tennessee, for the year 1882. W. F. Glenn, M.D., Nashville, President; C. C. Fite, M.D., Nashville, Secretary.

Fourth Annual Report of the State Board of Health of Illinois. For the year 1882. John H. Rauch, M.D., Springfield, Secretary.

